# LC-MS-based metabolomics revealed SLC25A22 as an essential regulator of aspartate-derived amino acids and polyamines in *KRAS*-mutant colorectal cancer

**DOI:** 10.18632/oncotarget.21093

**Published:** 2017-09-20

**Authors:** Xiaona Li, Arthur C.K. Chung, Shangfu Li, Lilan Wu, Jiaying Xu, Jun Yu, Chichun Wong, Zongwei Cai

**Affiliations:** ^1^ State Key Laboratory of Environmental and Biological Analysis, Department of Chemistry, Hong Kong Baptist University, Hong Kong, China; ^2^ Guangdong Provincial Academy of Chinese Medical Sciences, The Second Affiliated Hospital of Guangzhou University of Chinese Medicine, Guangdong, China; ^3^ Institute of Digestive Disease and Department of Medicine and Therapeutics, State Key Laboratory of Digestive Disease, Li Ka Shing Institute of Health Sciences, Chinese University of Hong Kong, Hong Kong, China; ^4^ Shenzhen Research Institute, The Chinese University of Hong Kong, Guangdong, China

**Keywords:** KRAS-mutant colorectal cancer, SLC25A22, LC-MS, metabolomics

## Abstract

SLC25A22, which encodes the mitochondrial glutamate transporter, is overexpressed in colorectal cancer (CRC) and is essential for the proliferation of CRC cells harboring *KRAS* mutations. However, the role of SLC25A22 on metabolic regulation in *KRAS*-mutant CRC cells has not been comprehensively characterized.

We performed non-targeted metabolomics, targeted metabolomics and isotope kinetic analysis of *KRAS*-mutant DLD1 cells with or without SLC25A22 knockdown using ultra-high-performance liquid chromatography (UHPLC) coupled to Orbitrap mass spectrometry (MS) or tandem MS (MS/MS).

Global metabolomics analysis identified 35 altered metabolites, which were attributed to alanine, aspartate and glutamate metabolism, urea cycle and polyamine metabolism. Targeted metabolomics including 24 metabolites revealed that most tricarboxylic acid (TCA) cycle intermediates, aspartate-derived asparagine, alanine and ornithine-derived polyamines were strongly down-regulated in SLC25A22 knockdown cells. Moreover, targeted kinetic isotope analysis showed that most of the ^13^C-labeled ornithine-derived polyamines were significantly decreased in SLC25A22 knockdown cells and culture medium. Exogenous addition of polyamines could significantly promote cell proliferation in DLD1 cells, highlighting their potential role as oncogenic metabolites that function downstream of SLC25A22-mediated glutamine metabolism.

Collectively, SLC25A22 acts as an essential metabolic regulator during CRC progression as it promotes the synthesis of aspartate-derived amino acids and polyamines in *KRAS* mutant CRC cells.

## INTRODUCTION

Colorectal cancer (CRC) is the third most common cancer worldwide [1]. *KRAS* oncogene, mutated in approximately 30%–50% of CRC patients [2], presents both as a prognostic and predictive marker for targeted therapy of CRC [3, 4], and mutations in *KRAS* results in non-response to anti-EGFR inhibitors [4–6]. In our previous study, we identified SLC25A22, which encodes a mitochondrial glutamate transporter, as a novel oncogene essential for the viability of CRC cell lines with simultaneous mutations in *APC* or *CTNNB1* and *KRAS* [7]. SLC25A22 enhances cell proliferation and survival by promoting the synthesis of glutamine-derived aspartate (Asp). Apart from its role in CRC tumorigenesis, SLC25A22 has also been found to be mutated in encephalopathies, which frequently involved in altering the highly conserved amino acids that will completely abolish glutamate carrier activity [8, 9]. However, the full spectrum of metabolic effects of SLC25A22 on *KRAS*-mutant CRC cell lines have not been comprehensively characterized.

Metabolomics analysis plays a crucial role in the discovery of potential metabolic biomarkers during development of drug and diagnosis, as well as in revealing gene function during cell metabolism [6, 11–13]. Global and targeted metabolomics, which respectively aims to profile the entire and specific components of the metabolome, provide signatures for various metabolic phenotype and aid in the understanding of the mechanism of action of drugs or genes in biological systems at the level of metabolites. Nuclear magnetic resonance (NMR) and mass spectrometry (MS) coupled to gas chromatography (GC) or liquid chromatography (LC) are mainly two approaches to identity and quantify the metabolome on a global scale [14, 15]. NMR detection allows rapid detection, small sample volumes and provides structural information. However, the sensitivity and dynamic range of NMR are lower than MS [16]. Due to its high sensitivity and reproducibility, LC-MS-based metabolomics is a promising strategy to further elucidate metabolic pathways regulated by SLC25A22 in *KRAS*-mutant CRC and provide insights into the therapy of CRC with *KRAS* mutation.

**Figure 1:**
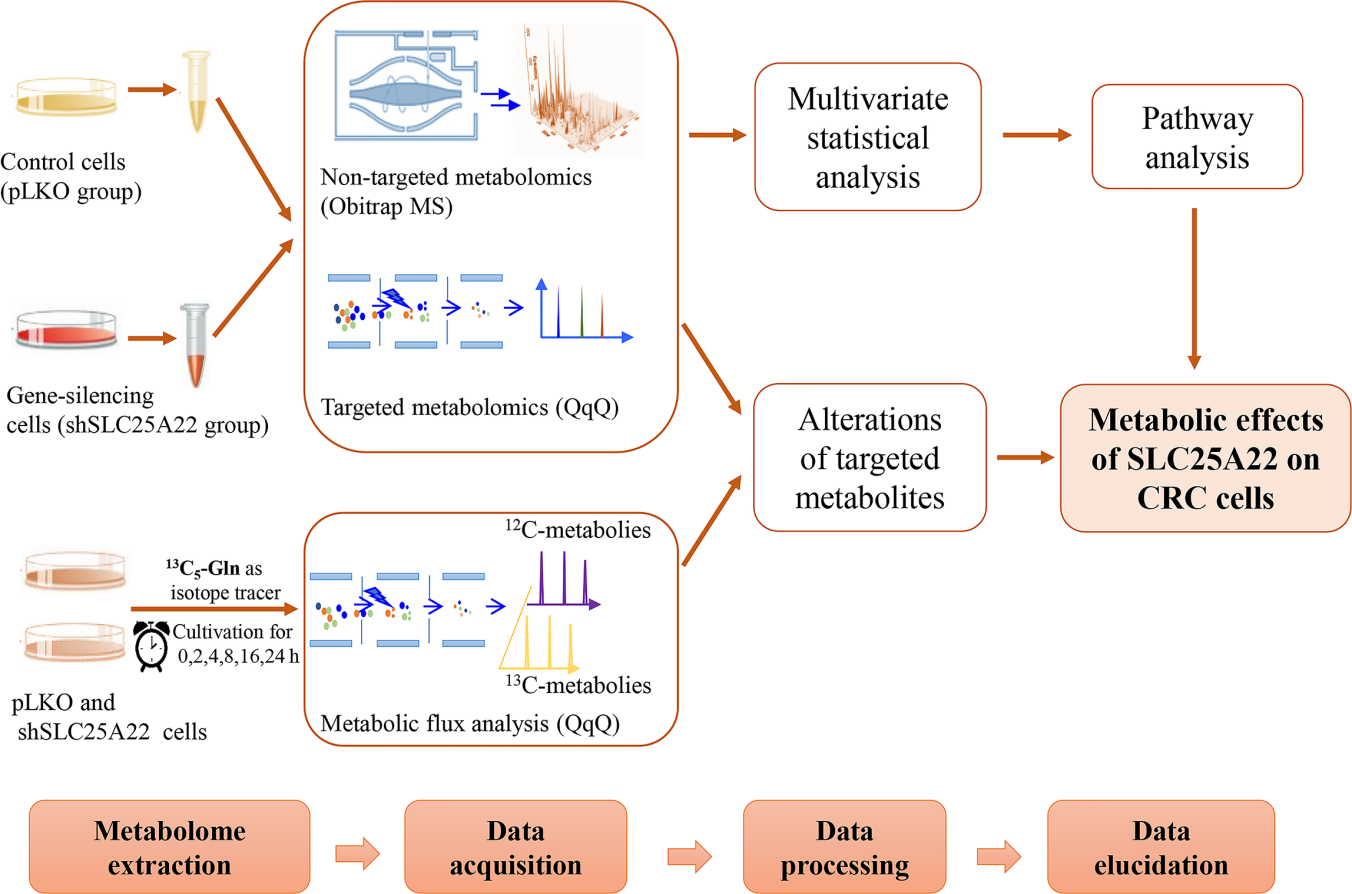
The workflow of global, targeted metabolomics analysis and kinetic isotope analysis

In the study, global and targeted metabolomics based on ultra-high-performance liquid chromatography coupled to mass spectrometry (UHPLC-MS) were utilized to evaluate the effects of SLC25A22 on cellular metabolism in *KRAS*-mutant CRC cells. The control (pLKO) and SLC25A22 knockdown (shSLC25A22) DLD1 cells were used. Moreover, targeted metabolomics analysis involving TCA cycle intermediates, amino acids and polyamines were conducted by triple-quadrupole (QqQ) MS. Finally, the metabolite fates of urea cycle intermediates and polyamines were traced via targeted kinetic analysis of DLD1 cells using [U-^13^C_5_]-glutamine as the isotope tracer. The workflow was shown in Figure 1. Our analyses revealed Asp-derived amino acids and polyamines as key oncogenic metabolites involved in SLC25A22-mediated cell proliferation in a CRC cell line with *KRAS*-mutation.

## RESULTS

### Global metabolomics analysis by UHPLC-Orbitrap-MS analysis

**Figure 2:**
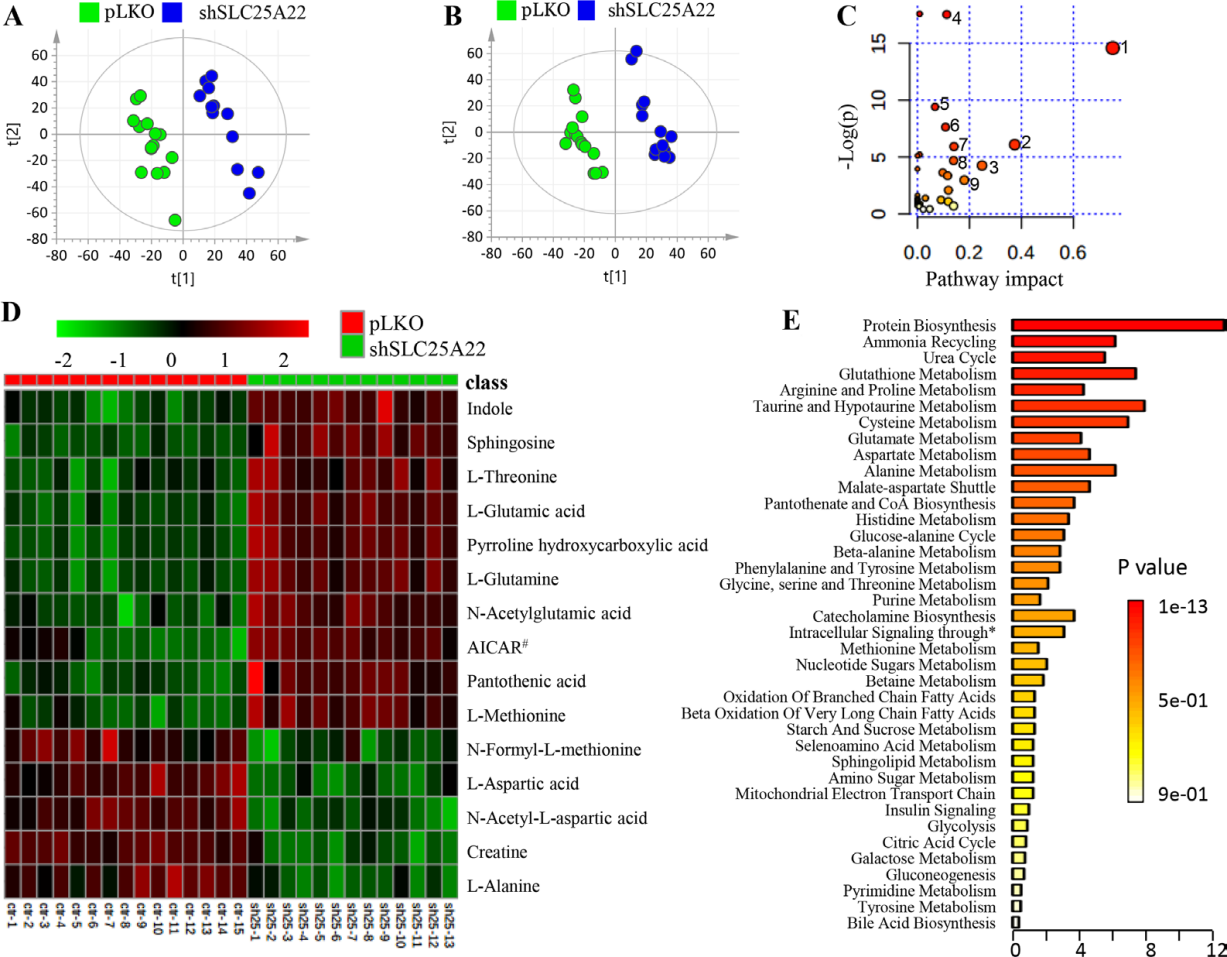
Global metabolic profiles of DLD1 cell line (*KRAS*-mutant CRC) stably expressing pLKO and shSLC25A22 (**A**) PLS-DA score plot in positive ion mode of LC-MS, showing clear separation between DLD1-pLKO cells (green dots) and DLD1-shSLC25A22 cells (blue dots). [t1]: component 1, [t2]: component 2. (**B**) PLS-DA score plot in negative ion mode. (**C**) Pathway analysis revealed that the silencing of SLC25A22 made the greatest impact on alanine, aspartate and glutamate metabolism. 1: Alanine, aspartate and Glutamate metabolism; 2: Taurine and hypotaurine metabolism; 3: Glutathione metabolism; 4: Aminoacyl-tRNA biosynthesis; 5: Cysteine and methionine metabolism; 6: Arginine and proline metabolism; 7: Histidine metabolism, 8: D-Glutamine and D-glutamate metabolism; 9: Pantothenate and CoA biosynthesis. (**D**) A Heatmap showed the top 15 significantly changed metabolites. AICAR^#^ represented 1-(5’-Phosphoribosyl)-5-amino-4-imidazolecarboxamide. (**E**) Enrichment analysis further showed top three important pathways, including protein biosynthesis, ammonia recycling and urea cycle. ^*^ represented intracellular signaling through prostacyclin receptor and prostacyclin.

In the global metabolomics analysis, a total of 6,195 and 5,260 metabolic features were obtained in positive and negative ion mode, respectively (total ion chromatogram (TIC) shown in Supplementary Figure 1A). Among them, 267 metabolites (175 in positive and 92 in negative mode) were identified. Eventually, we found 35 metabolites, out of which 16 were confirmed by comparing with authentic standards, were significantly altered with fold change (FC) of shSLC25A22/pLKO more than 1.1 or less than 0.8 (*t*-test: *p* < 0.05) through volcano plot screening (Supplementary Figure 1B and 1C) and VIP (variable importance in projection) over 1.0. The score plot in PLS-DA (partial least squares-discriminant analysis) model showed that shSLC25A22 cells were clearly separated from pLKO cells in positive and negative ion mode, respectively (Figure 2A and 2B).

Overall, specific sets of metabolites were altered considerably in shSLC25A22 cells compared to pLKO cells (*p* < 0.05), such as amino acids, nucleotides, carnitines, lipids, fatty acids, and their derivatives (Table 1). The pathway impact of alanine, aspartate and glutamate pathway over 0.6 demonstrated that metabolites, involving L-glutamine (Gln), L-glutamic acid (Glu), N-acetyl-aspartic acid (Ac-Asp), L-aspartic acid (Asp), L-asparagine (Asn) and L-alanine (Ala), made great contributions to the pathway analysis (Figure 2C). The heatmap of top 15 significantly changed metabolites was shown in Figure 2D. The FC of Gln and Glu were 1.33 (*p* = 2.6 e^−5^) and 1.29 (*p* = 1.1 e^−4^) between shSLC25A22 and pLKO, while the FC of Asp and Ac-Asp were of 0.55 (*p* = 2.4 e^−8^) and 0.71 (*p* = 5.7 e^−13^), indicating reduced conversion from Gln and Glu to Asp or Ac-Asp, which was partially consistent with previous reports [7, 17]. Furthermore, Asp-derived metabolites, particularly asparagine and alanine, were down-regulated in SLC25A22-silenced cells (Asn, FC = 0.79, *p* = 9.8 e^−5^; Ala, FC = 0.75, *p* = 4.2 e^−9^) as compared to DLD1-pLKO cells. AICAR (5-aminoimidazole-4-carboxyamide ribonucleoside) with the largest FC of 2.8 (*p* = 1.7 e-^6^) is an activator of AMP-activated protein kinase (AMPK) pathway, which inhibits cancer cell growth [18, 19]. Additionally, enrichment analysis further demonstrated that altered metabolites were involved in protein synthesis, ammonia recycling, urea cycle, glutamate metabolism, glutathione metabolism and aspartate metabolism (Figure 2E), which further confirmed the importance of alanine, aspartate and glutamate metabolism, polyamine and urea cycle metabolism.

**Table 1:** The list of identified metabolites changed significantly between shSLC25A22 and pLKO cell (in order of descending fold change)

No.	Compound name	m/z	RT/min	FC(s/p)^a^	*P*-value	MS Pattern	*∆ppm*	Mode	Pathway
1	AICAR^#^	339.0680	1.51	2.82	1.7E-06	110.0352, 97.0283, 127.0614	6.0	+/−	Histidine metabolism, purine metabolism
2	N-Acetylglutamic acid	190.0699	2.09	1.60	9.9E-06	190.0710, 172.0600, 130.0500	5.8	+/−	Biosynthesis of amino acids
3	Sphingosine	300.2879	13.38	1.54	2.4E-06	282.2783, 252.2679, 56.0497	5.9	+	Sphingosine metabolism
4	L-Glutamine^*^	147.0756	0.90	1.33	2.6E-05	147.0762, 130.0495, 84.0448	5.5	+	Alanine, aspartate and glutamate metabolism
5	Pyrroline hydroxycarboxylic acid	130.0492	0.91	1.31	9.2E-05	84.0447, 130.0493, 56.0505	5.2	+	Arginine and proline metabolism
6	L-Glutamic acid^*^	148.0595	0.92	1.29	1.1E-04	84.0446, 102.0547, 130.0497	6.3	+	Alanine, aspartate and glutamate metabolism
7	Glutathione^*^	308.0891	1.58	1.22	1.6E-02	308.0934, 233.0610, 162.0235	6.4	+/−	Glutathione metabolism
8	Indole	118.0644	6.05	1.21	6.9E-04	118.0648, 91.0540, 65.0386	6.0	+	Phenylalanine, tyrosine and tryptophan metabolism
9	L-Threonine^*^	120.0649	0.91	1.17	3.6E-02	56.0497, 74.0599, 102.0547	5.5	+	Aminoacyl-tRNA biosynthesis, threonine metabolism
10	Glycerophosphocholine	258.1086	0.91	1.15	1.4E-02	104.1067, 124.9995, 184.0729	5.8	+	Glycerophopholipid metabolism
11	L-Methionine^*^	150.0575	1.53	1.13	1.1E-02	150.0574, 133.0314, 104.0528	5.5	+	Aminoacyl-tRNA biosynthesis, Cysteine and methionine metabolism
12	Pantothenic acid^*^	220.1167	4.79	1.10	3.2E-02	90.0548, 220.1175, 202.1069	5.9	+/−	Pantothenate and CoA biosynthesis
13	Creatine^*^	132.0760	0.95	0.80	1.0E-10	132.0768, 90.0555, 87.0557	5.6	+/−	Arginine and proline metabolism
14	Butyrylcarnitine	232.1530	5.00	0.79	3.5E-03	232.1565, 173.0821, 85.0295	5.7	+	Fatty acid oxidation
15	L-Asparagine^*^	133.0599	0.90	0.79	9.8E-05	133.0602, 87.0551, 74.0242	6.8	+	Alanine, aspartate and glutamate metabolism
16	L-Histidine^*^	154.0612	0.86	0.78	1.2E-04	154.0613, 93.0446, 137.0346	6.5	−	Aminoacyl-tRNA biosynthesis, histidine metabolism
17	L-gamma-glutamyl-L-leucine	261.1430	5.56	0.78	8.2E-07	261.1450,198.1120, 132.1020	5.9	+	Biosynthesis of amino acids
18	Xanthine	151.0252	2.04	0.78	1.2E-02	151.0250, 108.0199	6.4	−	Purine metabolism
19	ADP^*^	426.0227	1.48	0.78	7.3E-03	426.0227, 158.9245, 78.9575	1.4	−	Purine metabolism
20	L-Tyrosine^*^	180.0659	4.28	0.77	5.0E-08	119.0490, 180.0659, 136.0757	3.9	−	Aminoacyl-tRNA biosynthesis, tyrosine metabolism
21	L-Phenylalanine^*^	164.0706	4.28	0.77	7.0E-08	147.0442, 164.0707, 72.0080	6.9	−	Aminoacyl-tRNA biosynthesis, phenylalanine metabolism
22	N-Acetyl-L-methionine	190.0538	5.68	0.77	1.7E-06	148.0428, 142.0499, 190.0537	2.7	−	Protein biosynthesis
23	N^1^, N^12^-Diacetylspermine^*^	287.2440	0.91	0.76	2.4E-02	100.0763, 171.1498	0.5		Polyamine metabolism
24	Gamma Glutamylglutamic acid	277.1014	1.51	0.76	5.5E-03	84.0442, 148.0600, 130.0496	5.9	+	Amino acids biosynthesis and metabolism
25	Uridine diphosphate glucose	565.0485	1.75	0.75	3.6E-02	323.0290, 565.0483, 384.9848	1.4	−	Nuclear sugar metabolism
26	N-Formyl-L-methionine	176.0380	5.31	0.75	3.5E-11	98.0234, 128.0342, 176.0379	4.1	−	Cysteine and methionine metabolism
27	L-Acetylcarnitine	204.1219	1.51	0.75	8.9E-08	85.0282, 204.1227, 60.0809	5.5	+	Fatty acid oxidation
28	L-Alanine^*^	90.0545	0.90	0.75	4.2E-09	90.0544	5.3	+	Alanine, aspartate and glutamate metabolism
29	Oxidized glutathione^*^	613.1558	2.43	0.74	5.0E-02	613.1592, 538.1252, 484.1162	5.7	+/−	Glutathione metabolism
30	N-Acetyl-L-aspartic acid	174.0399	1.01	0.71	5.7E-13	88.0390, 130.0499, 58.0282	5.1	−	Alanine, aspartate and glutamate metabolism
31	Gamma-Glutamyltyrosine	311.1219	4.76	0.69	8.5E-08	311.1240, 248.0920, 182.0810	6.0	+	Tyrosine metabolism
32	Uridine diphosphate-N-acetylglucosamine	606.0754	2.67	0.69	4.7E-04	606.0750, 384.9849, 282.0388	1.8	−	Nucleotide biosynthesis
33	L-Aspartic acid^*^	132.0291	0.90	0.55	2.4E-08	132.0291, 115.0025, 88.0399	8.7	−	Alanine, aspartate and glutamate metabolism
34	Taurine	126.0213	0.91	0.49	1.9E-06	126.0214, 108.0109	5.4	+/−	Taurine and hypotaurine metabolism
35	3-Sulfinoalanine	152.0013	0.92	0.48	1.9E-03	88.0390, 152.0017	6.7	−	Taurine and hypotaurine metabolism

Notes:

^a^FC (s/p) represented fold change between shSLC25A22 and pLKO cells.

^b^AAs and derivatives represented that amino acids and their derivatives.

^*^Represented the metabolite was identified by authentic standard and database, while the unmarked metabolite was identified by database.

^#^AICAR, 1-(5’-Phosphoribosyl)-5-amino-4-imidazolecarboxamide.

### Targeted metabolomics analysis by UHPLC-QqQ analysis

**Figure 3:**
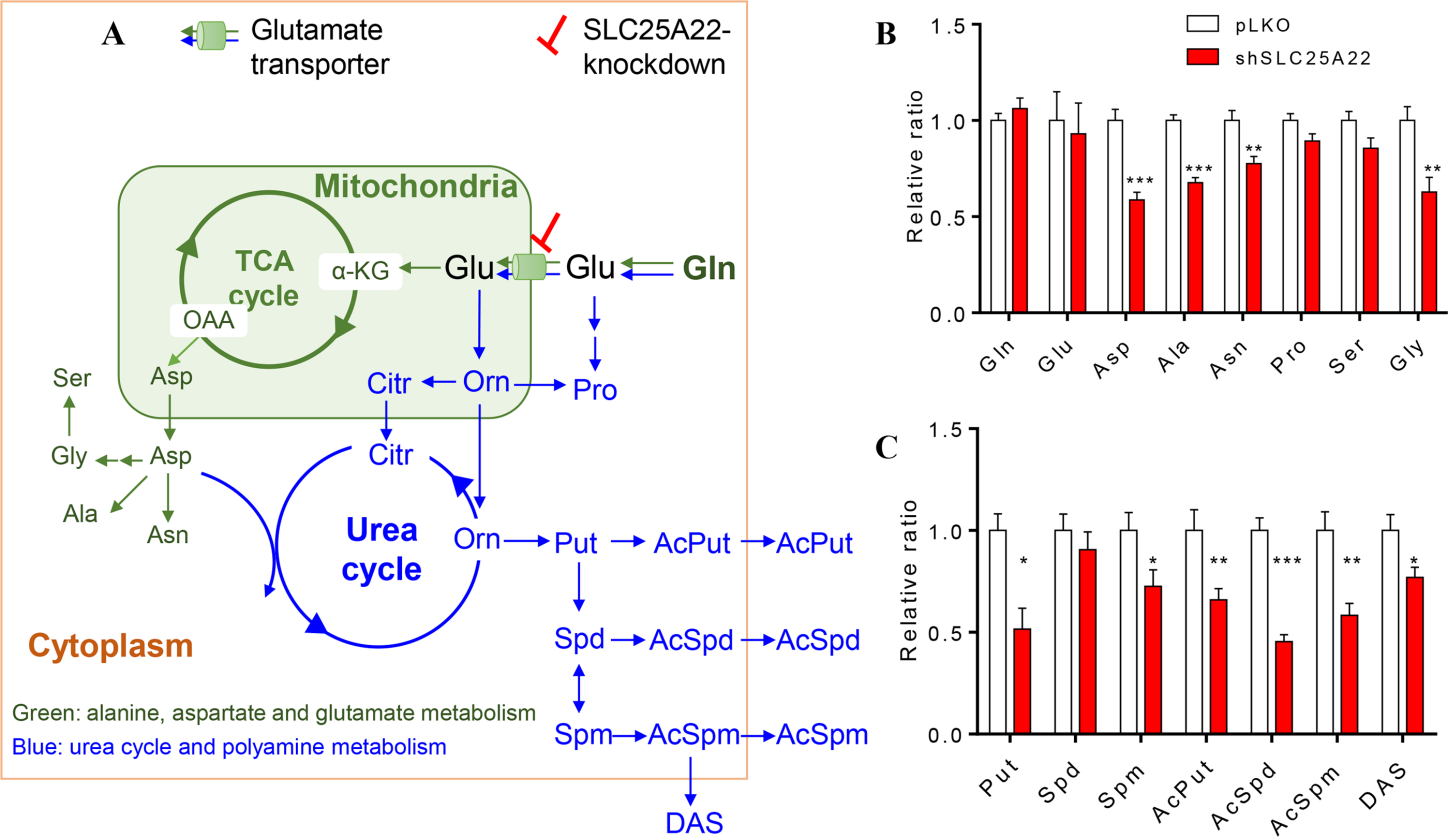
Targeted metabolomics metabolites between pLKO and shSLC25A22 cells (**A**) Scheme overview of glutamine metabolism in mitochondria and cytoplasm. The green arrows and metabolites represented the alanine, aspartate and glutamate metabolism, the blue arrows and metabolites represented the urea cycle and polyamine metabolism. (**B**) Relative ratio of amino acids in alanine, glutamate and aspartate pathway between pLKO and shSLC25A22 cells, (**C**) Relative ratio of polyamines between pLKO and shSLC25A22 cells. ^*^p < 0.05, ^**^p < 0.01, and ^***^p < 0.001. Error bar represented the SEM. Gln: glutamine, Glu, glutamate, α-KG: α-ketoglutarate, OAA: oxaloacetate, Asp: aspartate, Asn: asparagine, Ala: alanine, Pro: proline, Ser: serine, Gly: glycine, Orn: Ornithine, Citr: citrulline, Put: putrescine, Spd: spermidine, Spm: spermine, AcPut: *N^1^*-Acetylputrescine, AcSpd: *N^1^*-Acetylspermidine, AcSpm: *N*1-Acetylspermine, DAS: *N^1^, N^12^*-Diacetylspermine.

Gln serves as carbon and nitrogen source in cells. Gln is converted into Glu by removing one molecule of amide group, followed by entry into TCA cycle to generate Asp and Asp-derived amino acids, which belongs to the alanine, aspartate and glutamate metabolism (Figure 3A in green). On the other hand, Gln also participates in nitrogen metabolism including urea cycle and polyamine biosynthesis [20, 21] (Figure 3A in blue). Targeted analysis was thus performed on these metabolic pathways.

As shown in Supplementary Figure 2A, when the expression of SLC25A22 was knocked down in DLD1-shSLC25A22 cells, TCA cycle intermediates tended to be down-regulated, especially malate (*p* < 0.05) and fumarate (*p* < 0.05), which was in agreement with our previous study [7]. The relative ratio of up- and down-stream amino acids in alanine, aspartate and glutamate metabolism between shSLC25A22 cells and pLKO cells showed that Asp was close to 0.5 (*p* < 0.001), indicating strong reduction of this metabolite in SLC25A22 silenced cells. Moreover, levels of Ala, Asn and Gly were significantly reduced in shSLC25A22 cells, which were likely a consequence of reduced Asp levels (Figure 3B). Level of SLC25A22 expression in cells did not affect Gln, Glu, Ser and Pro differentially.

Urea cycle metabolites such as ornithine (Orn), citrulline (Citr) and arginine (Arg) were not significantly altered except for Asp, which functions as an intermediate to generate arginosuccinate (Supplementary Figure 2B). On the other hand, Orn-derived polyamines putrescine (Put, *p* < 0.05), spermine (Spm, *p* < 0.05) and acetylated polyamines, involving *N^1^*-acetylputrescine (AcPut, *p* < 0.01), *N^1^*-acetylspermidine (AcSpd, *p* < 0.001), *N^1^*-acetylspermine (AcSpm, *p* < 0.01), *and N^1^*, *N^12^*-diacetylspermine (DAS, *p* < 0.05) were suppressed in DLD1-shSLC25A22 cells (Figure 3C). These data indicated that knockdown of SLC25A22 expression profoundly affected TCA cycle and aspartate, an intermediate of urea cycle, whilst other urea cycle intermediates were not changed significantly, leading to reduced levels of TCA cycle intermediates, Asp-related amino acids and polyamines.

### Metabolic kinetic isotope analysis by UHPLC-QqQ-MS with [U-^13^C_5_]-glutamine as isotope tracer

**Figure 4:**
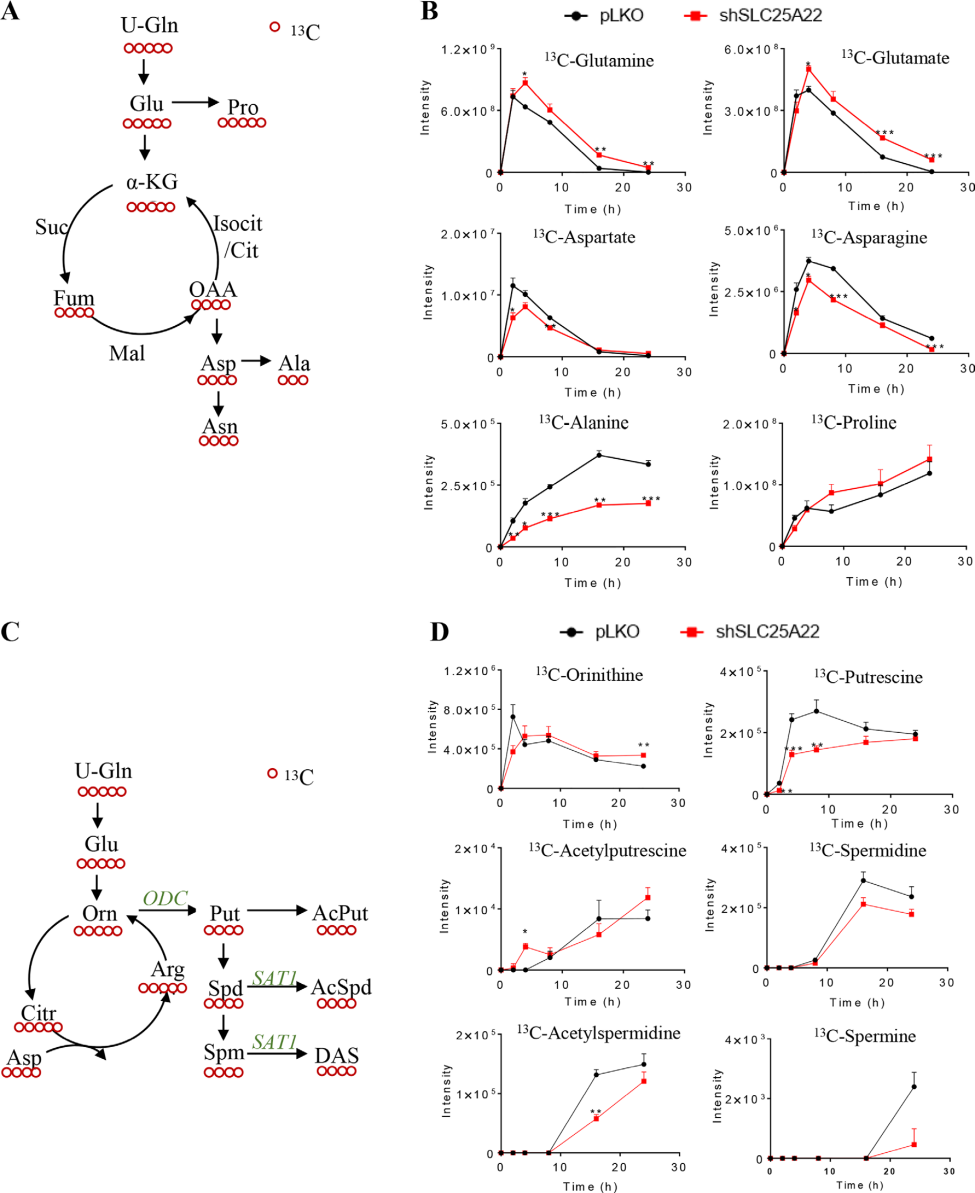
SLC25A22 knockdown inhibited polyamine biosynthesis but not triggered urea cycle in *KRAS*-mutant CRC cells (**A**) Schematic overview of metabolism of downstream ^13^C-labeled amino acids sourced from ^13^C_5_-glutamine; (**B**) Flux of ^13^C-Gln derived amino acids in shSLC25A22 and pLKO cells; (**C**) Schematic overview of metabolism in urea cycle and polyamines from ^13^C_5_-glutamine; (**D**) Flux of ^13^C-labeled urea cycle intermediates and ^13^C_4_-polyamines in shSLC25A22 and pLKO cells.

We next traced the metabolic fates of Asp-derived amino acids and polyamines using [U-^13^C_5_]-glutamine as isotope tracer. ^13^C_4_-Asp, ^13^C_4_-Asn and ^13^C_3_-Ala were labelled using [U-^13^C_5_]-Gln as carbon source (Figure 4A) [22]. Results demonstrated that ^13^C-labled Asp, Asn and Ala were significantly reduced in DLD1-shSLC25A22 cells in comparison with those in the control DLD1-pLKO cells at time points starting from 4 h (*p <* 0.05) (Figure 4B). Of note, the intensity of [U-^13^C_3_]-Ala in DLD1-shSLC25A22 cells at each time point was close to 0.5-fold compared to that in control DLD1-pLKO cells, indicating a strong reduction in the levels of this metabolite. Moreover, tendencies of ^12^C-metabolites were similar with ^13^C-metabolites (Supplementary Figure 3A and 3B), suggesting that SLC25A22-mediated glutamine metabolism is important for the biosynthesis of these amino acids.

The [U-^13^C_5_]-ornithine in urea cycle derived from [U-^13^C_5_]-Gln can be converted into [U-^13^C_4_]-Put via ornithine decarboxylase (*ODC*) and then further metabolized into Spd and Spm, which are acetylated and exported via passive diffusion across the cell membrane (Figure 4C) [23]. [U-^13^C_5_]-Orn were reduced in DLD1-shSLC25A22 cells compared with DLD1-pLKO cells before 2 h, while being up-regulated from 4 h to 24 h; however, other ^13^C_5_-urea cycle intermediates were not detected both in DLD1-pLKO and DLD1-shSLC25A22 cells (Figure 4D). On the other hand, all the ^13^C_4_-polyamine intermediates, with the exception of ^13^C_4_-AcPut at 4 h, were down-regulated in the SLC25A22 knockdown cells (^13^C_4_-AcSpm and ^13^C_4_-DAS were not detected). Of note, both ^12^C/^13^C-polyamines in the DLD1-shSLC25A22 cells and DLD1-shSLC25A22 conditioned cell culture media were significantly reduced compared to DLD1-pLKO cells (Supplementary Figure 3C and 3D). These data indicated that silencing of SLC25A22 significantly impaired the flux of Gln-derived carbons into polyamine biosynthesis.

### Western blot analysis and mRNA expression of polyamine biosynthetic enzymes

**Figure 5:**
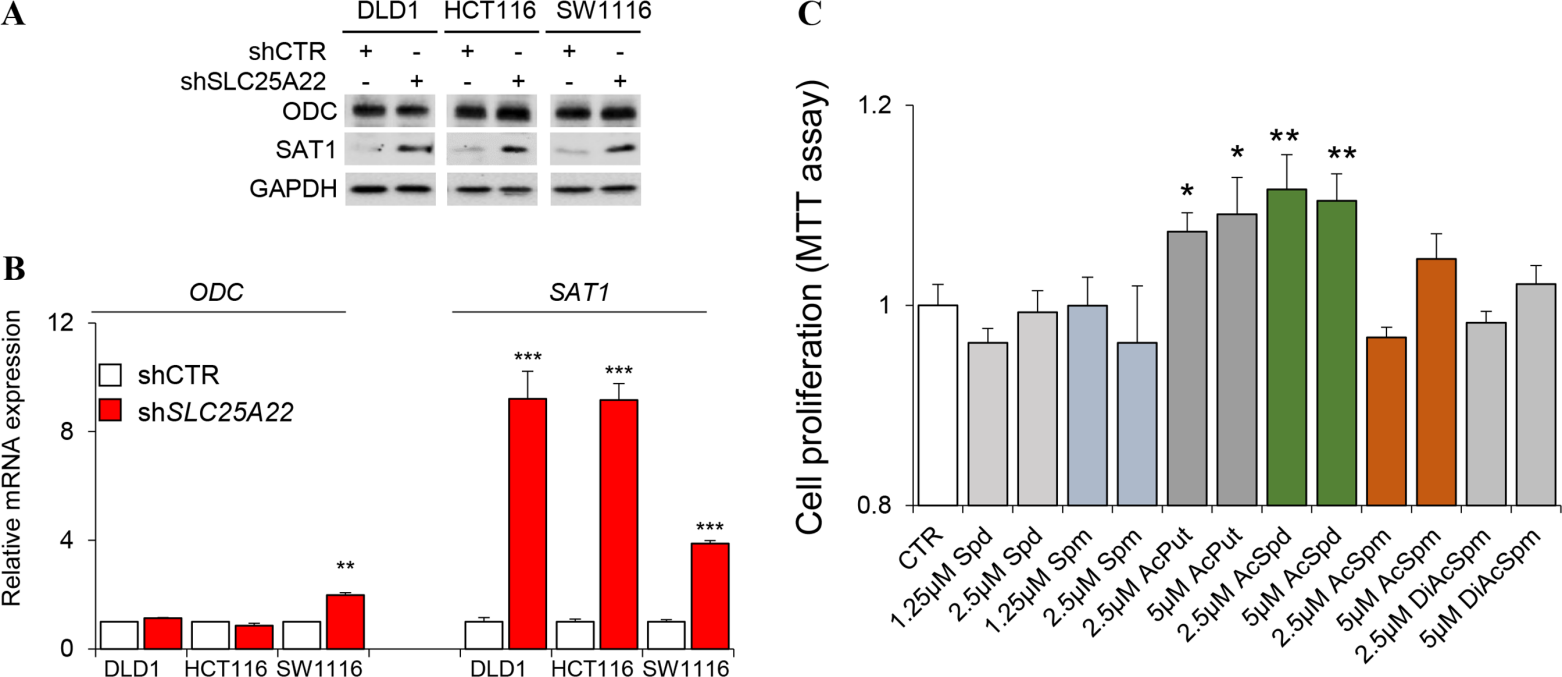
Western blot, qPCR and MTT analysis (**A**) Western blot of *ODC* and *SAT1* in shSLC25A22 and pLKO cells; (**B**) mRNA expression in qPCR analysis; (**C**) Cell proliferation in MTT assay. ^*^p < 0.05, ^**^p < 0.01, and ^***^p < 0.001. Error bar represented the SEM.

We validated whether the altered polyamine metabolism might be due to deregulated expression of enzymes involved in polyamine biosynthesis. However, western blot analysis revealed that *ODC* expression was not significant different in SLC25A22-silenced DLD1 cells; whilst expression of spermidine/spermine *N^1^*-acetyltransferase 1 (*SAT1*) was induced by knockdown of SLC25A22 (Figure 5A). Relative mRNA expression of ODC was not significantly different between control and SLC25A22-silenced cells in *KRAS*-mutant DLD1, HCT116, SW1116 cell line, while mRNA expression of *SAT1* was dramatically increased in SLC25A22-silenced cells compared with control cells (Figure 5B); which was consistent with the results of western blot. This suggests that the suppression of polyamines biosynthesis is likely a direct consequence of reduced glutamine flux, but not influenced by changes in gene expression. We thus speculated that induced *SAT1* expression might reflect an attempt to up-regulate polyamine metabolism in order to compensate for the reduced carbon flux from glutamine.

### Role of polyamines in the proliferation of *KRAS*-mutant CRC cells

Finally, we examined whether polyamines play a role in cell proliferation in *KRAS*-mutant CRC cells. We incubated DLD1 cells with six polyamines at 1.25/2.5 μM or 2.5/5 μM, and then examination cell viability by MTT assay. Our results showed that *N^1^*-AcPut and *N^1^*-AcSpd promoted CRC cell growth at both doses (Figure 5C).

## DISCUSSION

Due to the wide dynamic range and reproducible analysis, UHPLC-MS is a powerful tool for metabolomics and capable of systematic profiling of endogenous metabolites and uncovering the complex metabolic alterations that arise from gene mutation or aberrant gene expression [24, 25]. In the study, 15 DLD1-pLKO and 13 DLD1-shSLC25A22 cell extracts by chilled 80% MeOH were analyzed for global and targeted metabolomics analysis [26].

Compared shSLC25A22 cells to pLKO cells, global metabolomics analysis uncovered significantly altered amino acids were attributed to the alanine, aspartate and glutamate pathway. The findings were further confirmed by targeted analysis, which was complement with previous studies [7]. Some metabolites such as TCA cycle and urea cycle intermediates, nucleotides were found to be up-regulated in CRC patients [27, 28]. Particularly, specific amino acids were regulated differentially in colorectal tissues of cancer patients. Ala, Asp, Gly, Pro and Ser were up-regulated, whilst Gln and Glu were down-regulated in CRC compared with healthy controls [29]. Ala was reported to function as alterative carbon source that fuels tumor metabolism [30]. Asn was shown to be up-regulated in *KRAS*-mutant CRC via the overexpression of asparagine synthetase (ANSN) and it promotes protein biosynthesis in cancer cells by serving as an amino acid exchange factor regulates the uptake of amino acid and cell proliferation [31, 32]. Consistent with the oncogenic function of SLC25A22 in CRC, knockdown of SLC25A22 down-regulated the biosynthesis of TCA cycle metabolites and Asp-derived amino acids (Ala, Asp and Gly) that are up-regulated in CRC, which in turn, impair *KRAS*-mutant CRC cell growth.

Apart from these aforementioned metabolites, we unveiled that SLC25A22 knockdown had a profound impact on polyamine metabolism. Polyamines derived from ornithine are required for normal and cancer cells [23, 33], and their levels are frequently up-regulated in carcinogenesis. Johnson *et al*. found that polyamines, especially DAS as an end-product of polyamine metabolism, was strongly up-regulated in CRC tumor tissues compared to adjacent normal tissues using metabolomics approaches [13]. Here, our global metabolomics analysis showed that DAS was reduced in SLC25A22 knockdown cells (FC = 0.76, *P* = 2.4e^−2^). Consequently, targeted analysis on polyamines and urea cycle metabolites demonstrated that polyamines were remarkably decreased in DLD1-shSLC25A22 cells. However, urea cycle intermediates were not sufficiently labelled by [U-^13^C_5_]-glutamine to enable detailed kinetic analysis of the urea cycle, suggesting the urea cycle did not trigger by the knockdown of SLC25A22. Previous reports indicated that increased polyamine metabolism could enhance cancer growth, migration and metastasis [34, 35], while polyamines depletion could inhibit cancer cell proliferation, migration and invasion via SAT1 mediation [36]. Exogenous addition of some polyamine metabolites promoted growth of DLD1 cells, which confirmed their potential role as onco-metabolites in *KRAS*-mutant CRC. Taken together, SLC25A22-induced production of polyamines represents a novel mechanism whereby SLC25A22 mediates its oncogenic effect in *KRAS*-mutant CRC.

In summary, the data obtained through LC-MS-based global metabolomics, targeted metabolomics and kinetic isotope analysis indicated that SLC25A22 knockdown inhibited the biosynthesis of Asp-derived amino acids and polyamines in *KRAS*-mutant CRC cells. Furthermore, the addition of polyamine into culture medium can modulate CRC cell growth. Thus, our studies demonstrated that SLC25A22 is an essential regulator of the metabolic system of *KRAS*-mutant colorectal tumor and its overexpression promotes tumor cell growth, which could provide more insights into *KRAS*-mutant CRC therapy with treatment of polyamine-inhibitor.

## MATERIALS AND METHODS

### Chemicals and reagents

Pure water was prepared by Mili-Q system (Milipore, USA). Methanol (MeOH), acetonitrile (ACN), formic acid (FA), ammonium hydroxide and ammonium acetate were of LC grade. All standards of (D/L-) amino acids, putrescine, spermidine, spermine, *N^1^*-acetylputrescine and *N*^1−^acetylspermidine were purchased from Sigma (St. Lous, US). *N^1^*-acetylspermine, *N^1^, N^12^*-diacetylspermine were ordered from Cayman (MI, US). [U-^13^C_5_]-glutamine was obtained from Cambridge isotope laboratory (MA, US).

### Cell culture

DLD1 cell line was obtained from American Type Culture Collection (ATCC, Rockville, MD). All cells were routinely cultured in the Dulbecco's modified eagle's medium (DMEM) medium supplemented with 10% fetal bovine serum (FBS) and 100 unit/mL penicillin-streptomycin. DLD1 cells stably expressing pLKO (control, *n* = 15) and shSLC25A22 (SLC25A22 knockdown, *n* = 13) were cultured in the presence of puromycin (2 μg/mL). Cells were seeded at 5 × 10^6^ per 10 cm dish for 24 h prior to global and targeted metabolomics analysis.

For targeted kinetic isotope analysis, DLD1-pLKO and DLD1-shSLC25A22 cell lines were seeded at a density of 2 × 10^6^ cells/10 cm dish in complete DMEM in triplicates. After 24 h, the medium was replaced with glutamine-free MEM consisting of 2 mM [^13^C_5_]-glutamine. The cells were harvested at the following time points: 0 h, 2 h, 4 h, 8 h, 16 h and 24 h.

### Sample preparation

Culture medium was aspirated and the cells were washed twice in ice-cold PBS and once in pure water. The cells were extracted by adding 1 mL chilled methanol: H_2_O (v/v, 8:2) containing 0.1 μg/mL 4-chloro-phenylalanine (4-Cl-Phe) as the internal standard (IS). The cells were then incubated at -80 °C for 60 min, scraped and transferred into Eppendorf tubes. Cell extracts were subjected to three freeze-thaw cycles using liquid nitrogen and ice. The supernatant was transferred into a new tube after centrifugation for 10 min at 16000 *g*, 4°C. The residue was then extracted with 0.5 mL methanol, and the supernatants were pooled for lyophilization. The residues were stored at -80 °C prior to analyses.

Samples were reconstituted in 200 μL MeOH:H_2_O (v/v, 85:15); 100 μL was diluted to 50% MeOH (v/v) in water for non-targeted metabolomics study, followed by 30 μL pooled together from each participant as a quality control (QC) sample; and the other 100 μL for targeted metabolomics. All samples were detected within 24 h of reconstitution.

### Data acquisition of global metabolomics

The data was acquired from Ultimate 3000 rapid separation liquid chromatography (RSLC) coupled with Q Exactive Focus MS (Thermo Scientific, USA) for global metabolomics analysis. The Acquity UPLC HSS T3 (2.1 × 100 mm, 1.8 μm, Waters) was used to separate metabolites at 30°C. The mobile phases were water (A) and ACN (B), both with 0.1% FA (v/v). The injection volume was 10 μL. Data was acquired both in positive and negative ion mode. Specific LC-MS parameters were detailed in Supplemental Information. Cell samples were analyzed at random, and the sequence was performed in a “3 samples-1 QC” order after 3 QC samples. The QC sample was applied for analytical quality assurance and signal correlation [37].

### Data processing and metabolites identification of global metabolomics

The raw data from metabolic profiling acquired in UHPLC-Orbitrap-MS was first converted into CDF data format by using Xcalibur workstation (Thermo Scientific, USA), and metabolic features were extracted by running XCMS package under R version 3.2.2 with chromatographic alignment and matching [38]. The noise level of global metabolomics data of XCMS parameters was set at 50,000 in positive and 20,000 in negative ion mode, respectively. Subsequently, a three-dimensional csv-format document involving *m/z*, retention time (RT) and peak intensity were obtained. Next, data was filtered using “80% rule” [39], and normalized by the IS and protein content. Finally, the data was subjected to multivariate statistical analysis by SIMCA-P 13.0 (Umetrics, Sweden) after mean-centering and scaling to the standard deviation.

The potential biomarkers were identified by comparing exact *m/z*, retention time and MS/MS pattern of samples with those of authentic standards or those in database, such as Metlin (https://metlin.scripps.edu) and human metabolome database (HMDB, http://www.hmdb.ca) [40]. We applied 10 ppm as mass error and ± 6 s as retention time error for feature grouping and matching. Moreover, MS/MS pattern of potential biomarkers were collected at the resolution of 70,000, the isolation width of 0.6 amu, IT of 100 ms and the collision energies of 10, 20 and 30 eV. Pathway and enrichment analysis were conducted by MetaboAnalyst (http://www.metaboanalyst.ca/) [41].

### Targeted metabolomics and metabolic kinetic analysis

Metabolites analyzed by targeted metabolomics included TCA cycle intermediates, related amino acids and polyamines (SRM transitions were shown in Supplementary Table 1). The data were acquired by Ultimate 3000 RSLC coupled to TSQ Quantiva^TM^ triple-quadrupole MS (QqQ, Thermo Scientific, USA). The Xbridge BEH Amide column (2.1 × 100 mm, 1.7 μm, Waters) was used for metabolites separation at 30°C. Amino acids and polyamines were analyzed in positive ion mode, while the TCA cycle intermediates were analyzed in negative ion mode. Targeted kinetic isotope analysis was performed for polyamines and related amino acids of the TCA cycle and urea cycle. The SRM transitions involved ^12^C- or ^13^C-labeled metabolites were shown in Supplementary Table 2.

### Statistical analysis

In the global metabolomics analysis, differential metabolites between DLD1-pLKO cells and DLD1-shSLC25A22 cells were chosen by VIP over than 1 in the PLS-DA model and FC of shSLC25A22/pLKO more than 1.1 or less than 0.8 with significant differences (*p* < 0.05) in Student's *t*-test. Additionally, the data for targeted metabolomics and targeted kinetic isotope analysis were performed as described [17].

### Western blot

Total proteins were extracted using Cytobuster^TM^ Protein Extraction Reagent (EMD Millipore), denatured in loading buffer, and then separated by sodium dodecyl sulfate polyacrylamide gel electrophoresis (SDS-PAGE). Antibodies for *ODC* (ab66067) and *SAT1* (ab105220) were obtained from Abcam (Cambridge, MA).

### Quantitative PCR (qPCR)

Total RNA was extracted using Trizol reagent, and cDNA was synthesized using the High-Capacity cDNA Reverse Transcription Kit (Thermo Fisher Scientific, Waltham, MA). PCR was performed using FastStart Universal SYBR Green Master Mix (Roche, Basel, Switzerland) in a LC480 LightCycler (Roche). The following primer sequences were used: *ODC*, forward: 5′- TTCCAAAGCAGTCTGTCGTCT-3′ and reverse: 5′- GGAAGCTGACACCAACAACAT-3′; SAT1, forward: 5′-CCGTGGATTGGCAAGT TATT -3′ and reverse: 5′- TCCAACCCTCTTCACTGGAC-3′; and β-Actin, forward: 5′- AGAGCTACGAGCTGCCTGAC-3′ and reverse: 5′- AGCACTGTGTTGGCGTA CAG-3′.

### 3-(4,5-dimethylthiazol-2-yl)-2,5-diphenyltetrazolium bromide (MTT) assay

Cell growth curve was performed using MTT assay (Sigma-Aldrich, St. Louis, MO). Briefly, DLD1 cells were seeded into 96 well plates (1 × 10^3^ cells per well) overnight, followed by the addition of polyamines for 48 h. At the end of the incubation, MTT (0.5 mg/mL) was added to the medium for 4 h, and the reaction was stopped by the addition of 0.1 N HCl in 10% SDS. After overnight incubation, the plates are analyzed on a microplate reader (570 nm).

## SUPPLEMENTARY MATERIALS FIGURES AND TABLES



## Abbreviations

CRC: Colorectal cancer
UHPLC: ultra-high performance liquid chromatography
NMR: nuclear magnetic resonance
MS: mass spectrometry
TIC: total ion chromatogram
TCA: tricarboxylic acid
QqQ: triple-quadrupole
VIP: variable importance in projection
PLS-DA: partial least squares-discriminant analysis
Gln: glutamine
Glu: glutamate
α-KG: α-ketoglutarate
Suc: succinate
Fum: fumarate
Mal: malate
OAA: oxaloacetate
Cit: citrate
Isocit: isocitrate
Asp: aspartate
Asn: asparagine
Ala: alanine
Ser: serine
Gly: glycine
Orn: ornithine
Citr: citrulline
Arg: arginine
Pro: proline
AICAR: 1-(5′-Phosphoribosyl)-5-amino-4-imidazolecarboxamide
Orn: ornithine
Citr: citrulline
Arg: arginine
Put: putrescine
Spd: spermidine
Spm: spermine
AcPut: *N^1^*-acetylputrescine
AcSpd: *N^1^*-acetylspermidine
AcSpm: *N*1-acetylspermine
DAS: *N^1^, N^12^*-diacetylspermine
*ODC*: ornithine decarboxylase
*SAT1*: spermidine/spermine *N^1^*- acetyltransferase
ANSN: asparagine synthetase
4-Cl-Phe: 4-chloro-phenylalanine
IS: internal standard
SRM: selected reaction monitoring
RT: retention time
