# Supplementary material for: LC-MS-based metabolomics revealed SLC25A22 as an essential regulator of aspartate-derived amino acids and polyamines in KRAS-mutant colorectal cancer
Source: Oncotarget. 2017 Sep 20;8(60):101333–44. doi: 10.18632/oncotarget.21093 (PMC5731878; doi:10.18632/oncotarget.21093)
Supplement: Supplementary file 1 [file oncotarget-08-101333-s001.pdf]

LC-MS-based metabolomics revealed SLC25A22 as an essential regulator of aspartate-derived amino acids and polyamines in *KRAS*-mutant colorectal cancer

SUPPLEMENTARY MATERIALS

MATERIALS AND METHODS

LC-MS conditions in global metabolomics analysis

The LC gradient program was as follows: 0 min, 2% B; 1 min, 2% B; 19 min, 100% B; 21 min, 100 % B; 21.1 min, 2% B; 25 min, 2% B. The flow rate was 0.3 mL/ min. The QE Focus MS was equipped with a heated electrospray ionization (HESI) source. The MS parameters were as follows. The spray voltages were 3.5 kV for positive ion mode and 3 kV for negative ion mode, respectively. The pressure of sheath and auxiliary gas was set at 45 arb and 10 arb, respectively. The temperatures of capillary and auxiliary gas were both 320°C. The S-lens RF level was 60%. The scan range was 70–1000 (*m/z*). The resolution was 35,000. The maximum inject time (max IT) was 100 ms. Automated gain control (AGC) was set at 1 × e^6 ions.

LC-MS conditions in targeted metabolomics analysis (1)

The mobile phases used for the separation of TCA cycle intermediates, related amino acids and polyamines were water (A) and ACN (B) both containing 0.1% FA, while for the TCA cycle intermediates were water/ACN (A, v/v, 95:5, pH 9.45) containing 20 mM ammonium acetate and 20 mM ammonium hydroxide and acetonitrile (B). The flow rate was 0.3 mL/min. The LC gradient program was as follows: 0 min, 85% B; 12 min, 55% B; 14 min, 20% B; 16 min, 20% B; 16.5 min, 85 % B; 22 min, 85% B. The TSQ MS was also equipped with a heated electrospray ionization (HESI) source. The data were collected by using selected monitoring reaction (SRM) mode (Supplementary Table 1). The spray voltages were of 3.5 kV and 3 kV in positive and negative ion mode, respectively. The pressure of sheath and auxiliary gas was set at 30 arb and 10 arb. The temperature of capillary and auxiliary gas was 320°C and 320°C. The CID gas was set at 1.5 mTorr.

Supplementary Table 1: SRM transitions of TCA cycle intermediates, polyamines and related amino acids in targeted metabolomics analysis by LC-QqQ-MS

	Metabolite	Abbreviations	Polarity	Precursor Ion	Product Ion	CE[a] (V)
TCA cycle Intermediates	Citrate	Cit	Negative	191	87	25
	Isocitrate	Isocit	Negative	191	73	25
	α-ketoglutarate	α-KG	Negative	145	101	8
	Succinate	Suc	Negative	117	73	15
	Fumarate	Fum	Negative	115	71	10
	Malate	Mal	Negative	133	115	12
	Oxaloacetate	OAA	Negative	131	87	15
	Aspartate	Asp	Negative	132	88	15
	4-Cl-Phenylalanine	ISb	Negative	198	181	15
Related amino acids	Glutamate	Glu	Positive	148	84	15
	Glutamine	Gln	Positive	147	84	10
	Asparagine	Asn	Positive	133	74	15
	Alanine	Ala	Positive	90	44.3	10
	Proline	Pro	Positive	116	70	18
	Ornithine	Orn	Positive	133	70	20
	Citrulline	Citr	Positive	176	70	10
	Arginine	Arg	Positive	175	70	15
Polyamines	Putrescine	Put	Positive	89.1	72.1	12
	N^1-Acetylputrescine	AcPut	Positive	131.1	114.2	12
	N^1-Acetylputrescine	AcPut	Positive	131.1	72.1	18
	Spermidine	Spd	Positive	146.1	72.1	15
	Spermidine-2	Spd	Positive	146.1	112.1	15
	N^1-Acetylspermidine	AcSpd	Positive	188.2	72.1	20
	N^1-Acetylspermidine-2	AcSpd	Positive	188.2	100.1	18
	Spermine	Spm	Positive	203.2	112.1	20
	Spermine-2	Spm	Positive	203.2	129.1	12
	N^1-Acetylspermine	AcSpm	Positive	245.2	129.2	15
	N^1-Acetylspermine-2	AcSpm	Positive	245.2	112.2	20
	N^1,N^{12}-diacetylspermine	DAS	Positive	287.2	100.1	25
	N^1,N^{12}-diacetylspermine-2	DAS	Positive	287.2	171.2	18
	IS		Positive	200	154	15

Notes: [a]represents collision energy, [b]represented internal standard.

Supplementary Table 2: SRM transitions of polyamines and amino acids in isotope kinetic analysis by LC-QqQ-MS

Class	Metabolite	Polarity	Precursor Ion	Product Ion	CE (V)
	Gln	Positive	147	84	20
	$^{13}C_5$-Gln	Positive	152	88	20
	Glu	Positive	148	84	15
	$^{13}C_5$-Glu	Positive	153	88	15
	Asp	Positive	134	74	15
Related amino acids	$^{13}C_4$-Asp	Positive	138	76	15
	Asn	Positive	133	74.2	15
	$^{13}C_4$-Asn	Positive	137	76	15
	Ala	Positive	90	44.3	10
	$^{13}C_3$-Ala	Positive	93	46.3	10
	Pro	Positive	116	70	18
	$^{13}C_5$-Pro	Positive	121	74	18
	Orn	Positive	133.1	70	20
	$^{13}C_5$-Orn	Positive	138	74	20
	Arg	Positive	175	70.2	15
Urea cycle amino acids	$^{13}C_5$-Arg	Positive	180	74	15
	Citr	Positive	176	159.1	10
	$^{13}C_5$-Citr	Positive	181	163.1	10
	IS	Positive	200	154	15
	Put	Positive	89.1	72.1	12
	Put-$^{13}C_4$	Positive	93.1	76.1	12
	AcPut	Positive	131.1	114.2	12
	AcPut-2	Positive	131.1	72.1	18
	AcPut-$^{13}C_4$*	Positive	135.1	118.1	12
	AcPut-$^{13}C_4$-2	Positive	135	76.1	18
	Spd*	Positive	146.1	72.1	15
	Spd-2	Positive	146.1	112.1	15
	Spd-$^{13}C_4$*	Positive	150.1	76.1	15
	Spd-$^{13}C_4$-2	Positive	150.1	116.1	15
	AcSpd*	Positive	188.2	72.1	20
	AcSpd-2	Positive	188.2	100.1	18
Polyamines	AcSpd-$^{13}C_4$*	Positive	192.2	76.1	20
	AcSpd-$^{13}C_4$-2	Positive	192.2	104.1	18
	Spm*	Positive	203.2	112.1	20
	Spm-2	Positive	203.2	129.1	12
	Spm-$^{13}C_4$*	Positive	207.2	116.1	20
	Spm-$^{13}C_4$-2	Positive	207.2	133.1	12
	AcSpm*	Positive	245.2	112.2	20
	AcSpm-2	Positive	245.2	129.2	15
	AcSpm-$^{13}C_4$*	Positive	249.2	116.2	20
	AcSpm-$^{13}C_4$	Positive	249.2	133.2	15
	DAS*	Positive	287.2	100.1	25
	DAS-2	Positive	287.2	171.2	18
	DAS-$^{13}C_4$*	Positive	291.2	104.1	25
	DAS-$^{13}C_4$-2	Positive	291.2	175.2	18

Notes:
[a]represents collision energy, * represented quantitative transition, while the other transition was qualitative transition.

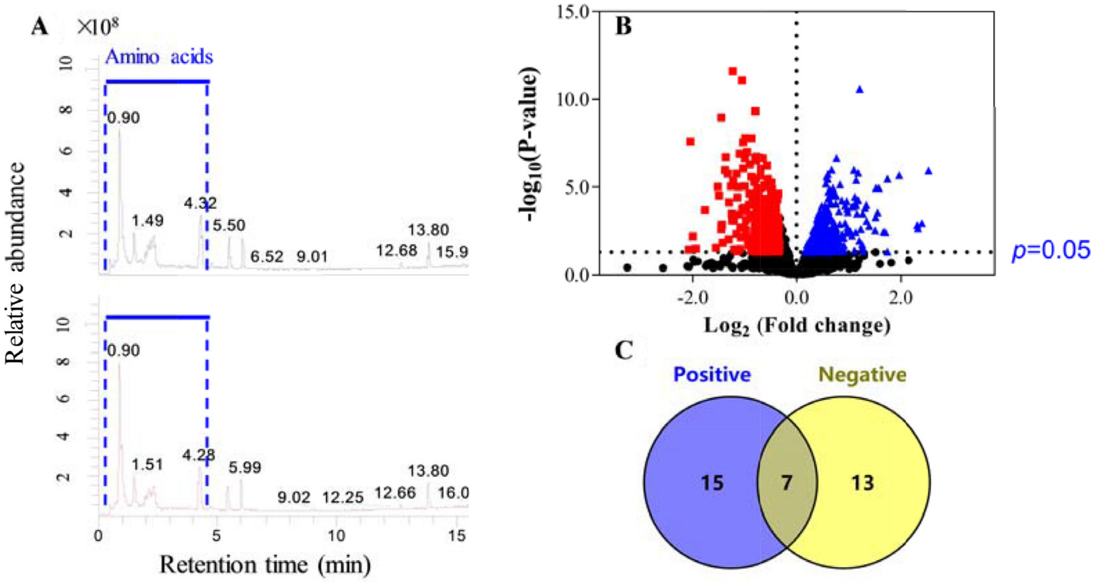

Supplementary Figure 1: The total ion chromatogram (TIC), volcano plot and Venn diagram of changed metabolites. (**A**) The total ion chromatogram (TIC) of control (pLKO, upper) and knockdown of SLC25A22 (shSLC25A22, lower) cells in positive ion mode of UHPLC-MS. (**B**) Volcano plot of global metabolomics data in positive mode. The X-axis was plotted on log2 scale of fold change (FC) of shSLC25A22/pLKO, while the Y-axis plotted the -log10 scale of *p*-value. The blue triangle represented metabolic features with FC less than 0.8 and *p*-value less than 0.05, and the red square represented metabolic features with FC over than 1.1 and *p*-value less than 0.05. (**C**) Venn diagram of identified metabolites both in positive and negative ion mode of UHPLC-MS. Twenty-two and 20 metabolites were found in positive and negative mode of LC-MS, respectively; and 7 metabolites were simultaneously detected in two modes

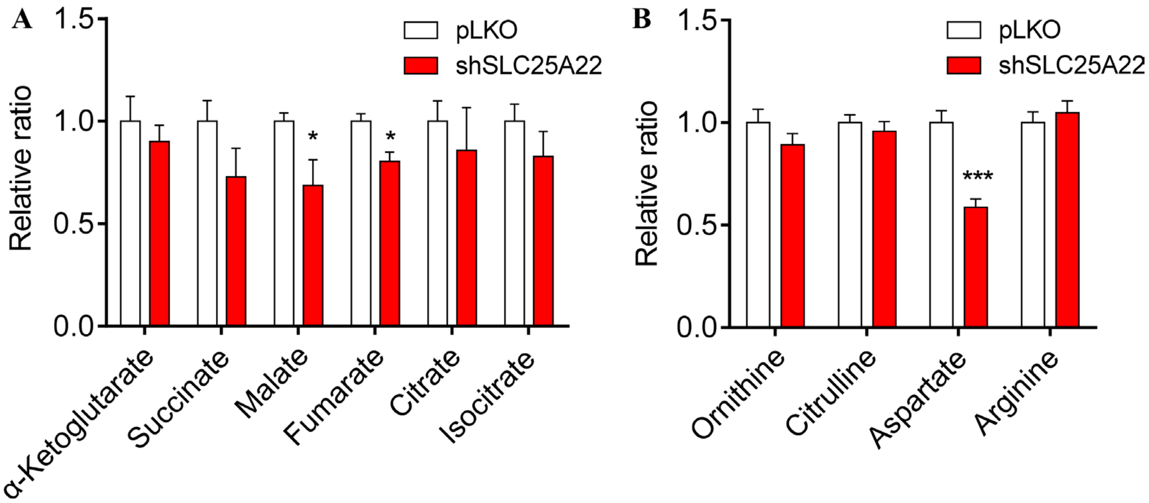

Supplementary Figure 2: Relative ratio of targeted metabolites between pLKO and shSLC25A22 cells. (**A**) TCA cycle intermediates, (**B**) urea cycle metabolites. $^{*}p < 0.05$, $^{**}p < 0.01$, and $^{***}p < 0.001$. Error bar represented the SEM.

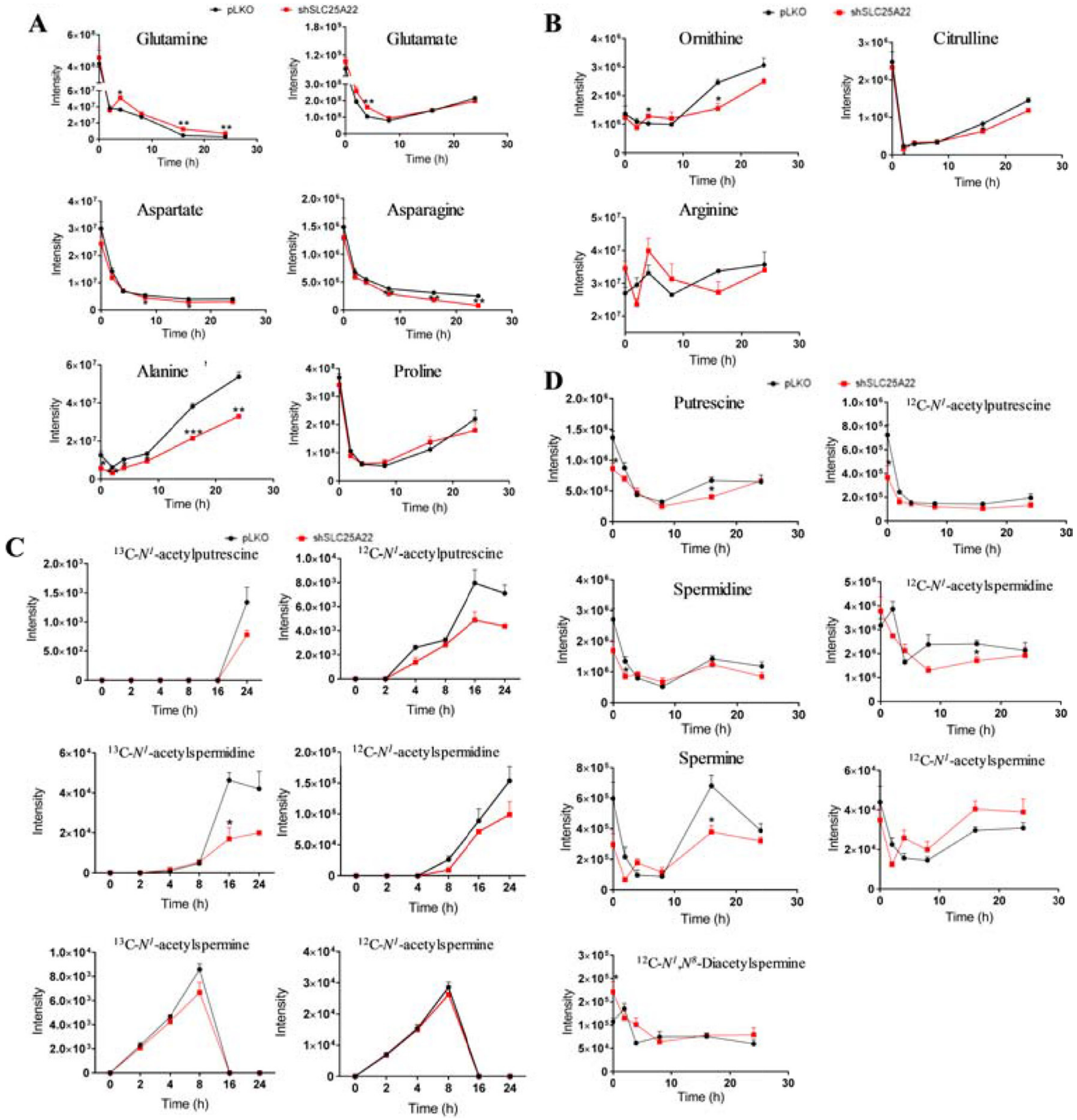

Supplementary Figure 3: Kinetic isotope analysis between pLKO and shSLC25A22 cells. (**A**) Flux of native amino acids related to TCA cycle intermediates in DLD1 cells; (**B**) Flux of native urea cycle intermediates in DLD1 cells; (**C**) Flux of ^{13}C-labeled and native acetylated polyamines in medium; (**D**) Relative ratio of native polyamines in DLD1 cells. $^{*}p < 0.05$, $^{**}p < 0.01$, and $^{***}p < 0.001$. Error bar represented the SEM.

REFERENCES

1. Li X, et al. Determination of amino acids in colon cancer cells by using UHPLC-MS/MS and [U-$^{13}C_5$]-glutamine as the isotope tracer. Talanta. 2017; 162:285–292.
